# Regulation of split anergy in natural killer cells by inhibition of cathepsins C and H and cystatin F

**DOI:** 10.18632/oncotarget.4208

**Published:** 2015-06-13

**Authors:** Špela Magister, Han-Ching Tseng, Vickie T. Bui, Janko Kos, Anahid Jewett

**Affiliations:** ^1^ Jožef Stefan Institute, Department of Biotechnology, 1000 Ljubljana, Slovenia; ^2^ The Jane and Jerry Weintraub Center for Reconstructive Biotechnology, Division of Oral Biology and Medicine, UCLA School of Dentistry, University of California-Los Angeles, Los Angeles, CA, USA; ^3^ University of Ljubljana, Faculty of Pharmacy, 1000 Ljubljana, Slovenia; ^4^ The Jonsson Comprehensive Cancer Center, UCLA School of Dentistry and Medicine, University of California-Los Angeles, Los Angeles, CA, USA

**Keywords:** NK cells, anergy, cathepsins C, cathepsin H, cystatin F

## Abstract

Freshly isolated human primary NK cells induce preferential lysis of Oral Squamous Carcinoma Stem Cells (OSCSCs) when compared to differentiated Oral Squamous Carcinoma Cells (OSCCs), while anti-CD16 antibody and monocytes induce functional split anergy in primary NK cells by decreasing the cytotoxic function of NK cells and increasing the release of IFN-γ. Since NK92 cells have relatively lower levels of cytotoxicity when compared to primary NK cells, and have the ability to increase secretion of regulatory cytokines IL-10 and IL-6, we used these cells as a model of NK cell anergy to identify and to study the upstream regulators of anergy. We demonstrate in this paper that the levels of truncated monomeric cystatin F, which is known to inhibit the functions of cathepsins C and H, is significantly elevated in NK92 cells and in anergized primary NK cells. Furthermore, cystatin F co-localizes with cathepsins C and H in the lysosomal/endosomal vesicles of NK cells. Accordingly, the mature forms of aminopeptidases cathepsins C and H, which regulate the activation of effector granzymes in NK cells, are significantly decreased, whereas the levels of pro-cathepsin C enzyme is increased in anergized NK cells after triggering of the CD16 receptor. In addition, the levels of granzyme B is significantly decreased in anti-CD16mAb and target cell anergized primary NK cells and NK92 cells. Our study provides the cellular and molecular mechanisms by which target cells may utilize to inhibit the cytotoxic function of NK cells.

## INTRODUCTION

Natural killer (NK) cells are large granular lymphocytes that mediate natural immunity against variety of tumor and virally infected cells [1]. Many activating and inhibitory NK cell receptors for target cell recognition have been elucidated in recent years, and the functional properties of NK cells have been the subject of many studies previously. For instance, NK cells have been shown to lose cytotoxic function following their interaction with target cells [2, 3]. Cytotoxicity pathways appear to be dissociated from proliferation and secretion of cytokines in NK cells [4]. This response termed ˝split anergy˝ has been observed in subsets of NK cells, NK_DC_ (NK cells dissociated from tumor conjugates) and NK_C_ (NK cells not dissociated from the tumor conjugate). Whereas NK_DC_ responded to IL-2 activation and become cytotoxic, they were unresponsive to IL-2 mediated induction of proliferation or secretion of cytokines. In contrast, NK_C_ showed an inverse response namely, they did not display any cytotoxicity following IL-2 activation, but proliferated and secreted cytokines [4, 5]. Treatment of NK cells with IL-2 and anti-CD16 antibody also induced split anergy [6–8]. Furthermore, IL-2 rescued anti-CD16 antibody mediated apoptosis induced in a subset of NK cells [8].

Many factors responsible for the tumor associated suppression of NK cell cytotoxicity have been identified, including the over-expression of Fas-ligand, and down-regulation of granzyme B [9] and CD16 receptor and its associated zeta chain [10–12]. However, the mechanisms by which tumor cells contribute to the induction of NK cell suppression are complex and have not been fully understood. Several human cancer cell types exhibit constitutively activated nuclear factor kappa B (NFκB) [13]. NFκB activity in tumor cells has been shown to have an inhibitory effect on the NK cell function as the inhibition of NFκB functional activity by the upstream super-repressor IκB increases the activation of NK cell cytotoxicity [14, 15]. It is also known that certain subsets of the myeloid arm of the immune system, such as monocytes, induce resistance in tumor cells and suppress NK cell mediated cytotoxicity [16–18]. Myeloid derived suppressor cells were shown to inhibit the function of NK cells in hepatocellular carcinoma in part via NKp30 receptors [19]. Elevated TGF-β1 and down-modulation of NKG2D have been shown to contribute to NK cell suppression in cancer patients [20]. It was also found that melanoma mediated inhibition of expression of NKp30, NKp44, and NKG2D, with subsequent impairment of NK cell–mediated cytolytic activity against melanoma cell lines was due to the functions of Indoleamine 2, 3-dioxygenase (IDO) and prostaglandin E2 (PGE2) [21].

Cysteine cathepsins are lysosomal cysteine peptidases that are involved at different levels of the innate and adaptive immune responses [22–24]. Cathepsin C is synthesized as pro-enzyme with molecular mass of 55 kDa, which is processed into mature cathepsin C [25]. Mature cathepsin C consists of a part of the pro-region, also known as residual pro-part, and of the catalytic region [25–27]. The catalytic part is cleaved into a heavy and light chain of 24 kDa and 6 kDa, respectively, and the active enzyme exists as a tetramer [25, 28]. Amino-dipeptidase cathepsin C functions as a key enzyme in the activation of granule serine peptidases, granzymes A and B, in cytotoxic T lymphocytes and NK cells [29]. Granule exocytosis includes the release of a pore-forming protein perforin and activated granzymes, into the synaptic region formed between the killer and the target cell [30, 31]. Granzyme B is the most potent pro-apoptotic molecule in the granule-mediated death of target cells [32]. It is synthesized as a pro-granzyme B with an inhibitory dipeptide and upon reaching the secretory lysosomes it is activated by cathepsin C [33, 34]. Although cathepsin C generates the majority of granzyme B activity, some studies support alternative mechanisms for processing and activation of granzyme B. For instance, lymphocytes derived from patients with congenital deficiency of cathepsin C (Papillon-Lefèvre syndrome) contain active granzyme B and kill target cells with the efficiency similar to healthy controls [35]. Similarly, the lymphocytes from cathepsin C-null mice display reduced but still appreciable granzyme B activity and kill target cells almost as efficiently as lymphocytes from wild-type mice [36]. Recently, an alternative pro-granzyme B convertase was suggested to be cathepsin H [37]. The processing of cathepsin H pro-peptide is an autocatalytic, multistep process proceeding from an inactive 41 kDa pro-form, through a 30 kDa intermediate form, to the 28 kDa mature form [38]. Mature 28 kDa form contains an additional octapeptide, termed the mini-chain, which originates from the pro-peptide and is bound to the mature enzyme by a disulfide bond and is essential for the aminopeptidase activity of cathepsin H [39].

The activity of cysteine cathepsin is controlled by their endogenous inhibitors, cystatins. Cystatins comprise a superfamily of evolutionarily related proteins, each consisting of at least one inhibitory domain of 100–120 amino acid residues [40–42]. Type I cystatins, or stefins, are cytosolic proteins, type II cystatins are predominantly secreted from the cells, whereas type III cystatins, the kininogens, are multifunctional proteins found in blood and other body fluids [43]. Cystatins are considered to be typical emergency inhibitors, trapping proteases escaped from the endosomes/lysosomes or cells in stable, proteolytically inactive complexes [42] and are thus generally considered not to regulate protease activity within the endosomal/lysosomal pathway. However, cystatin F is an exception. Although it is a secretory type II cystatin, it is present intracellularly to a much greater degree than other type II cystatins [44] and furthermore, it is localized in endosomal/lysosomal vesicles [45, 46] due to mannose 6-phosphate targeting pathway [47]. Cystatin F possesses unique features among human type II cystatins [48–50]. As it shares only 35% sequence identity with other members, has a 6 amino acid extension at the N terminus and is one of only two glycosylated type II cystatins [50]. In contrast to other family members, it has two additional cysteines (Cys26 and Cys63) and forms intermolecular disulfide bonds with another cystatin F molecule [50, 51]. Cystatin F is produced in cells as a disulfide-linked dimer [52], inactive as an inhibitor of cysteine cathepsins [45]. *In vitro*, unusually strong reducing conditions are needed to dissociate dimer to monomer [45]. However, the truncation of the N-terminal region to Lys35, presumably by cathepsin V [53] significantly enhances the monomerization and also changes the inhibitory properties of the monomer [54]. Intact monomeric cystatin F was shown to bind tightly cysteine endopeptidases such as cathepsins L, F, K and V, less tightly cathepsins S and H, but not exopeptidases cathepsins B, X and C [45, 48, 50]. By N-terminal truncation, cystatin F becomes a strong inhibitor of cathepsin C [54] but weaker inhibitor of cathepsin S, whereas the inhibitory potential towards cathepsin H was only slightly increased [46].

In this study we investigated the relationship between expression and activity of cathepsins C and H, their endogenous inhibitor cystatin F and cytotoxicity of primary NK cells. First, we demonstrated that primary NK cells are capable of lysing cancer stem cells and much less of differentiated tumors, and that the lysis is significantly decreased when treated with anti-CD16 antibody. We also demonstrate that the effect of monocytes on NK cells is similar to that of anti-CD16 antibody or tumor-target mediated induction of split anergy. We show that triggering CD16 antigen on NK cells decreases the expression of mature cathepsins C and H, affecting the activation of effector granzymes. At the same time the level of cystatin F increases, additionally suppressing cathepsins C and H activity. The results presented in this paper suggest a role for cystatins and cathepsins in bifurcation of cytotoxicity and cytokine secretion in NK cells.

## RESULTS

### Anti-CD16 antibody inhibited untreated and IL-2 treated NK cell lysis of both primary human differentiated Oral Squamous Carcinoma Cells (OSCCs) and Oral Squamous Carcinoma Stem Cells (OSCSCs), whereas it increased secretion of IFN-γ by the NK cells

**Figure 1:**
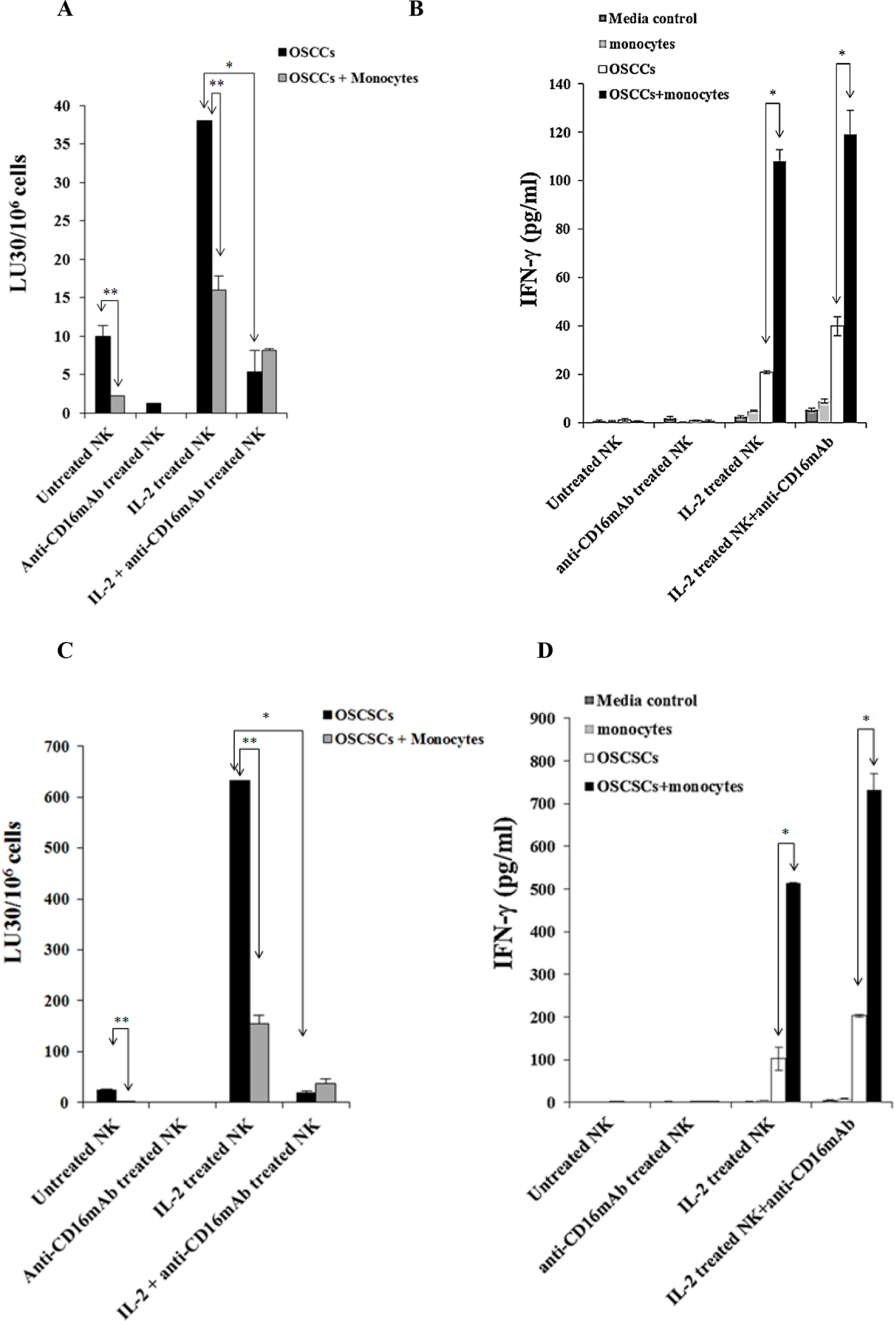
Monocytes protected primary differentiated Oral Squamous Carcinoma Cells (OSCCs) and Oral Squamous Carcinoma Stem Cells (OSCSCs) against NK cell mediated cytotoxicity, but significantly augmented the secretion of IFN-γ in co-cultures of NK cells, monocytes and tumor cells OSCCs **A.** or OSCSCs **C.** at 1 × 10^6^ cells/plate were co-cultured with and without irradiated monocytes (10 Gy) (monocytes: tumor cells ratio of 1:1) for 24–48 hours before they were removed from the plates, washed and labeled with ^51^Cr and used as targets in the cytotoxicity assays against NK cells. The NK cells from different donors were either left untreated or treated with anti-CD16mAb (3 μg/ml), IL-2 (1000 units/ml), or a combination of IL-2 (1000 units/ml) and anti-CD16mAb (3 μg/ml) for 24–48 hours before they were added to ^51^Cr labeled OSCCs or OSCSCs at different effector to target (E:T) ratios. Supernatants were removed after 4 hours of incubation and the released radioactivity counted by a γ counter. % cytotoxicity was determined at different E:T ratio, and LU30/10^6^ cells were calculated using the inverse of the number of effectors needed to lyse 30% of the tumor cells × 100. Minimum one of twenty representative experiments is shown for each cell in this figure. *The difference between IL-2 activated NK cells with OSCCs or OSCSCs and IL-2+anti-CD16mAb treated NK cells with OSCCs or OSCSCs is significant at *p* < 0.05. **The difference between untreated or IL-2 treated NK cells cultured with OSCCs or OSCSCs with and without monocytes is significant at *p* < 0.05. 1 × 10^5^ OSCCs **B.** or OSCSCs **D.** were co-cultured with and without irradiated monocytes at 1:1 ratio (OSCCs or OSCSCs:monocytes) for 24–48 hours before untreated or IL-2 (1000 units/ml) pre-treated or anti-CD16mAb (3 μg/ml) pre-treated, or a combination of IL-2 (1000 units/ml) and anti-CD16mAb (3 μg/ml) pre-treated NK cells at 1:1:1 ratios (NK:monocyte:tumor) were added. NK cells were pre-treated as indicated for 24–48 hours before they were added to the cultures of monocytes with tumors. After 24–48 hours of the addition of NK cells the supernatants were removed from the cultures and the levels of IFN-γ secretion were determined using a specific ELISA. Minimum one of twenty representative experiments is shown for each tumor type in this figure. *The difference between IL-2 activated NK cells incubated with OSCCs or OSCSCs and those of IL-2 treated NK cells cultured with OSCCs or OSCSCs with monocytes or IL-2+anti-CD16mAb treated NK cells cultured with and without OSCCs or OSCSCs with monocytes is significant at *p* < 0.05.

As shown in a number of previous studies [6, 8, 55] and in this report, anti-CD16mAb treatment induced anergy in NK cells, thereby inhibiting NK cell cytotoxicity against different target cells including K562 cells. In this study, two types of patient derived tumors, OSCCs and OSCSCs were tested for their sensitivity to NK cell mediated cytotoxicity and ability to induce IFN-γ secretion by NK cells. Treatment of NK cells with anti-CD16mAb decreased cytotoxicity against both tumor types (Figures 1A and 1C). The addition of IL-2+anti-CD16mAb also significantly inhibited the NK cell cytotoxicity against both tumor cell lines when compared to IL-2 activated NK cells (*p* < 0.05) (Figures 1A and 1C). Untreated or anti-CD16mAb treated NK cells did not secrete IFN-γ when co-cultured with any of the tumor cell populations but did so when treated with IL-2 and with IL-2 in combination with anti-CD16mAb (*p* < 0.05) (Figures 1B and 1D). In addition, both types of tumor cell lines triggered higher secretion of IFN-γ from IL-2+anti-CD16mAb treated NK cells when compared to IL-2 treated NK cells (Figures 1B and 1D).

### Monocytes protected primary human differentiated OSCCs and OSCSCs against NK cell mediated cytotoxicity and induced significant secretion of IFN-γ by the NK cells

The addition of monocytes to primary human differentiated OSCCs or OSCSCs prior to cytotoxicity assay inhibited the NK cell mediated lysis of OSCCs (Figure 1A) or OSCSCs (Figure 1C). Significant inhibition of NK cell cytotoxicity by monocytes can be observed against untreated or IL-2 treated NK cells against both tumor types (*p* < 0.05) (Figures 1A and 1C). These data indicate that monocytes protect differentiated OSCCs and stem-like OSCSCs against NK cell mediated lysis.

As expected IL-2 treated NK cells when co-cultured with OSCCs or OSCSCs secreted higher amounts of IFN-γ (Figures 1B, 1D). The addition of anti-CD16mAb in combination with IL-2 to NK cells cultured with OSCCs or OSCSCs increased secretion of IFN-γ when compared to IL-2 alone treated NK cells (Figures 1B and 1D). Monocytes added to IL-2 alone or IL-2+anti-CD16mAb treated NK cells in the presence of OSCCs or OSCSCs synergistically increased the levels of secreted IFN-γ compared to NK cells without monocytes (Figures 1B and 1D).

### Lack of cytotoxic function and decreased secretion of IFN-γ, TNF-α and GM-CSF, and increased secretion of IL-10 and IL-6 by NK92 cells when cultured with and without OSCSCs and OSCCs

**Figure 2:**
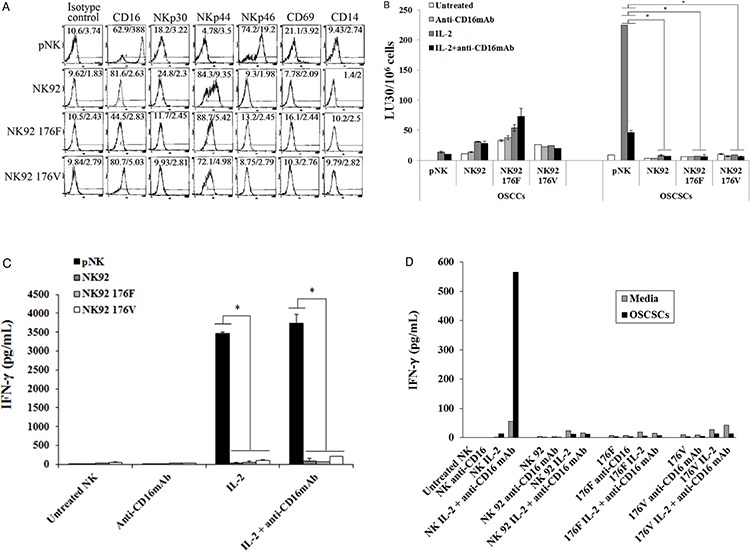

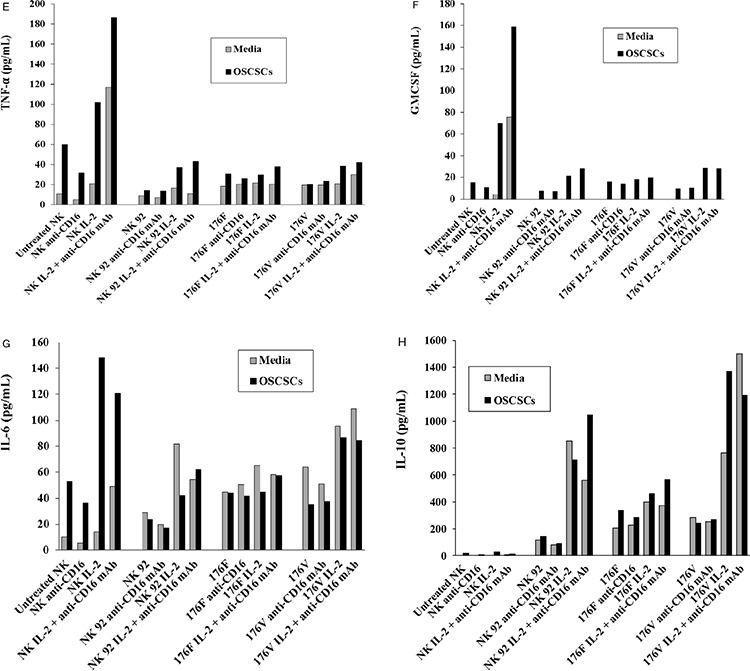
NK92 cells mediated no/low cytotoxicity or no/low secretion of IFN-γ when compared to primary NK cells The surface expression of CD16, NKp30, NKp44, NKp46, CD69 and CD14 on primary donor derived NK cells or NK92 parental line and its CD16 high (176V) and low (176F) variant transfectants were assessed after staining with the PE conjugated antibodies and analyzed using flow cytometry. Isotype control antibodies were used as controls. The numbers on the right hand corner are the percentages and the mean channel fluorescence intensities for each histogram **A.** NK cells or NK92 parental cells with its transfectants (1 × 10^6^ cells/ml) were left untreated or treated with anti-CD16mAb (3 μg/ml), IL-2 (1000 units/ml), or a combination of IL-2 (1000 units/ml) and anti-CD16 antibody (3 μg/ml) for 18–24 hrs. After which primary human NK cells and NK92 cells were added to ^51^Cr labeled OSCCs or OSCSCs at different E:T ratios. NK cell cytotoxicity was determined using a standard 4 hour ^51^Cr release assay and the lytic units 30/10^6^ cells were determined using inverse number of NK cells required to lyse 30% of the target cells × 100. Minimum one of five representative experiments is shown in this figure. *The differences between IL-2 or IL-2+anti- CD16mAb treated primary NK cells and those mediated by IL-2 or IL-2+anti-CD16mAb treated NK92 cells or its transfectants is significant at *p* < 0.05 **B.** Primary NK cells (1×10^5^/ml) and those of NK92 cells (1×10^5^/ml) and its transfectants were activated as described in Figure 2B, and after overnight incubation the supernatants were collected and the levels of IFN-γ were determined using specific ELISA. Minimum one of eight representative experiments is shown. *The differences between IL-2 or IL-2+anti-CD16mAb treated primary NK cells and those of IL-2 or IL-2+anti-CD16mAb treated NK92 cells or its transfectants is significant at *p* < 0.05 **C.** Highly purified primary NK cells (1×10^5^/ml) and NK92 cells (1×10^5^/ml) and its CD16 transfectants (1×10^5^/ml) were treated as described in Figure 2B and cultured without and with OSCSCs at an effector to target ratio of 0.5 to 1 for 24 hours. Afterwards, the supernatants were removed from the co-cultures and the levels of IFN-γ **D.** TNF-α **E.** GM-CSF **F.** IL-6 **G.** and IL-10 **H.** were determined by multiplexed Luminex analysis. One of three representative experiments is shown in this figure.

**Figure 3:**
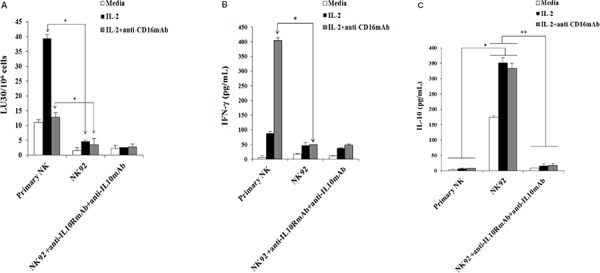

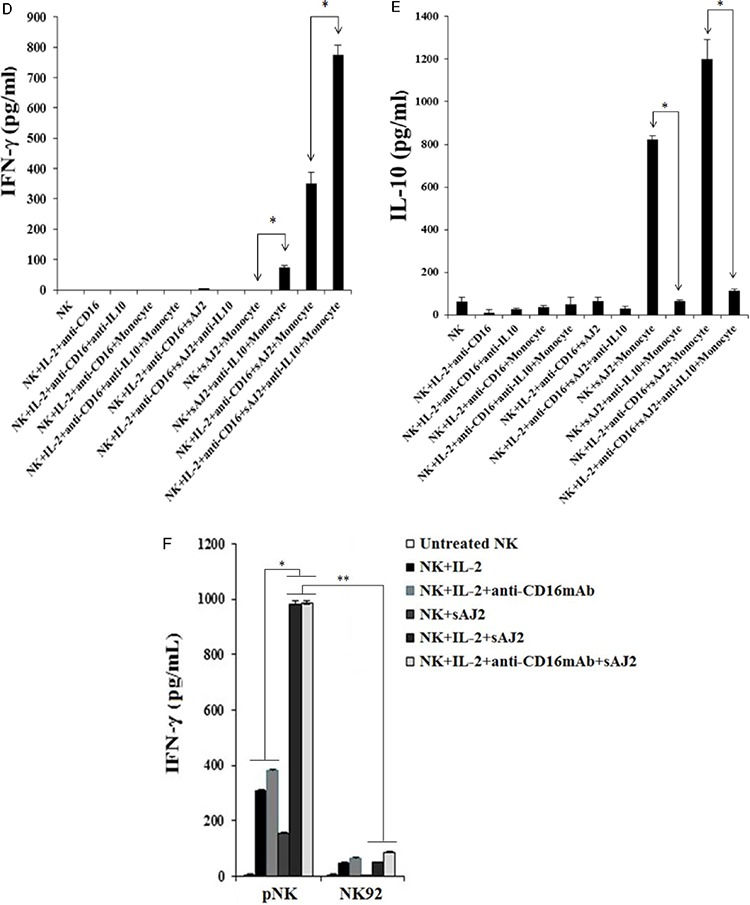
Blocking IL-10 in NK92 cells did not increase cytotoxicity or IFN-γ secretion One million purified primary NK cells or NK92 parental cells were left untreated or treated with IL-2 (1000 units/ml) or the combination of IL-2 (1000 units/ml) and anti-CD16mAb (3 ug/ml) in the presence and absence of anti-IL-10mAb (10 μg/ml) and anti-IL-10RmAb (5 μg/ml). After an overnight incubation, treated primary human NK cells and NK92 cells were added to ^51^Cr labeled OSCSCs at different E:T ratios. NK cell cytotoxicity was determined using a standard 4 hour ^51^Cr release assay, and the lytic units 30/10^6^ cells were determined using inverse number of NK cells required to lyse 30% of the target cells × 100. Minimum one of five representative experiments is shown in this figure. *The differences between IL-2 or IL-2+anti-CD16mAb treated primary NK cells and those mediated by IL-2 or IL-2+anti-CD16mAb treated NK92 cells is significant at *p* < 0.05. No significant differences could be obtained between untreated, IL-2 and/or anti-CD16mAb treated NK92 cells in the presence and absence of anti-IL-10mAb and anti-IL-10RmAbs **A.** Primary NK cells (1×10^5^/ml) and NK92 (1×10^5^/ml) were treated as described in Figure 3A. Afterwards, the supernatants from each of the NK cell samples or NK92 cells were removed and the levels of IFN-γ **B.** and IL-10 **C.** were determined using specific ELISAs. *The differences between IL-2+anti-CD16mAb treated primary NK cells and those mediated by IL-2+anti-CD16mAb treated NK92 cells is significant at *p* < 0.05 for IFN-γ, and the differences between untreated, IL-2 treated and IL-2+anti-CD16mAb treated primary NK cells and untreated, IL-2 treated and IL-2+anti-CD16mAb NK92 cells is significant at *p* < 0.05 for IL-10 secretion. No significant differences could be obtained for NK92 cells treated with isotype control antibody or anti-IL-10mAb and anti-IL-10RmAbs for IFN-γ secretion, whereas **p < 0.05 was obtained for differences of untreated, IL-2 treated and IL-2+anti-CD16mAb treated NK92 cells between isotype control treated and those of anti-IL-10mAb and anti-IL-10RmAbs for IL-10 secretion. Minimum one of five representative experiments is shown. Untreated, and IL-2 (1000 units/mL) +anti-CD16mAb (3 μg/ml) treated primary NK cells (1×10^5^/ml) were co-cultured with and without monocytes and sAJ2 at (1:1:2, NK:monocyte:bacteria) in the presence or absence of anti-IL-10mAb (10 μg/ml) for 12–18 hours before the supernatants were collected and the level of IFN-γ secretion were determined using IFN-γ ELISA. Minimum one of three representative experiments is shown. *The difference between NK cells cultured with sAJ2+monocytes or IL-2+anti-CD16mAb+sAJ2+monocytes and those treated with either sAJ2+monocytes+anti-IL10 mAb or IL-2+anti-CD16mAb+sAJ2+monocytes+anti-IL-10 mAb are significant at *p* < 0.05 **D.** NK cells were treated and co-cultured with and without monocytes and sAJ2 as described in Figure 3D in the presence or absence of anti-IL-10mAb (10 μg/ml) for 12–18 hours before the supernatants were collected and the level of IL-10 secretion was determined using specific ELISA. Minimum one of three representative experiments is shown in this figure. *The difference between NK cells cultured with sAJ2+monocytes or IL-2+anti-CD16mAb+sAJ2+monocytes and those treated with either sAJ2+monocytes+anti-IL10 mAb or IL-2+anti-CD16mAb+sAJ2+monocytes+anti-IL-10 mAb are significant at *p* < 0.05 **E.** Primary NK cells (1×10^5^/ml) and NK92 cells (1×10^5^/ml) were left untreated or treated with IL-2 (1000 units/ml), or IL-2 (1000 units/ml) +anti-CD16mAb (3 μg/ml) with or without sAJ2 (1:2; NK:bacteria) and the secretion of IFN-γ was determined using specific ELISA. Minimum one of three representative experiments is shown. *The differences between IL-2 or IL-2+anti-CD16mAb with or without sAJ2 is significant at *p* < 0.05. **the difference between primary NK cells treated with IL-2 and/or anti-CD16mAb+sAJ2 and NK92 cells treated with IL-2 and/or anti-CD16mAb+sAJ2 are significant at *p* < 0.05 **F.**

The function of primary NK cells was compared to NK92 parental line and its CD16 high and low variant transfectants (Figure 2). As shown in Figure 2A primary untreated NK cells expressed very high levels of CD16 and NKp46 and much lower levels of NKp30 and no expression of NKp44, whereas NK92 cells expressed much lower levels of CD16 receptor and the levels were moderately increased when CD16 expression was determined on high affinity CD16 transfectant (Figure 2A). Unlike primary NK cells, no expression of NKp46 could be seen on all three NK92 cells whereas they expressed significant levels of NKp44 (Figure 2A). No expression of CD69 or CD14 surface receptors could be seen on either primary NK cells or NK92 cell lines (Figure 2A). To assess cytotoxicity mediated by primary NK cells and those mediated by NK92 parental and its CD16 transfectants, we compared the lysis of OSCSCs by untreated, anti-CD16mAb, IL-2 and IL-2+anti-CD16 antibody treated primary NK cells and NK92 cells using ^51^Cr release assay. Unlike primary NK cells, NK92 parental and its CD16 transfectants were unable to lyse OSCSCs with and without treatment with IL-2 and/or anti-CD16mAb (*p* < 0.05) (Figure 2B). As expected, the addition of anti-CD16mAb antibody to NK cells inhibited cytotoxicity mediated by IL-2 treated primary NK cells (*p* < 0.05) (Figure 2B). IL-2 treated primary NK cells secreted significantly high levels of IFN-γ (*p* < 0.05) and the levels plateaued when NK cells were treated with IL-2+anti-CD16mAb, whereas no/very low secretion of IFN-γ could be obtained either with NK92 parental cells or its CD16 transfectants under different treatment conditions (Figure 2C). To determine the release of cytokines from primary NK cells and NK92 cells when cultured with and without OSCSCs, NK cells were treated with and without anti-CD16mAb, IL-2 and IL-2+anti-CD16mAb before they were cultured with OSCSCs. Treatment of primary NK cells with IL-2+anti-CD16mAb resulted in a significant increase in the production of IFN-γ (Figure 2D), TNF-α (Figure 2E), GM-CSF (Figure 2F) and IL-6 (Figure 2G). The levels of secretion synergistically increased when IL-2+anti-CD16mAb treated primary NK cells were co-incubated with OSCSCs. The treatment of IL-2 in primary NK cells resulted in a slight increase in cytokine secretion, and the levels were lower compared to IL-2+anti-CD16mAb treated NK cells (Figures 2C, 2D, 2E and 2F). NK92 cells treated with IL-2 produced no/low levels of IFN-γ and TNF-α (Figures 2C and 2E). Unlike primary NK cells, NK92 cells treated with IL-2+anti-CD16mAb did not result in a significant change in the secretion of IFN-γ (Figure 2C and 2D), TNF-α (Figure 2E) and GM-CSF (Figure 2F). However, IL-2 treated NK92 cells secreted higher levels of IL-6 and IL-10 when compared to untreated NK92 cells or untreated and IL-2 treated primary NK cells, and the amounts by NK92 cells generally decreased or remained similar when cultured in the presence of OSCSCs (Figures 2G and 2H). Addition of IL-2 with anti-CD16mAb to NK92 cells decreased the secretion of IL-6 and IL-10, and the levels were generally decreased or remain similar when co-cultured with OSCSCs (Figures 2G and 2H). To determine whether increase in IL-10 was responsible for the decreased cytotoxicity and IFN-γ secretion in NK92 cells, we added anti-IL-10mAb in the presence of IL-10RmAb and assessed the levels of cytotoxicity (Figure 3A) IFN-γ secretion (Figure 3B). Addition of anti IL-10mAb with IL-10RmAb was not able to increase cytotoxicity or IFN-γ secretion in NK92 cells even though it completely blocked the secretion of IL-10 (*p* < 0.05) (Figures 3A and 3C) whereas in primary NK cells it was able to increase IFN-γ secretion when added to untreated or IL-2+anti-CD16mAb treated NK cells cultured with monocytes and sAJ2, a combination of 8 probiotic bacteria (*p* < 0.05) (Figure 3D). Blocking of IL-10 in primary NK cells with antibodies to IL-10 was confirmed with ELISA (*p* < 0.05) (Figure 3E). IL-2 or IL-2+anti-CD16mAb treated NK92 cells cultured with sAJ2 did not change IFN-γ secretion when compared to IL-2 or IL-2+anti-CD16mAb treated primary NK cells (Figure 3F). Therefore, although NK92 cells may moderately respond to IL-2 and up-regulate IFN-γ and TNF-α, these cells have lost ability to respond to CD16 cross linking, as well as response to sAJ2. Transfection of CD16 receptor in NK92 cells did not restore the ability of IL-2+anti-CD16mAb to upregulate IFN-γ and TNF-α. Thus, NK92 and primary NK cells may share upstream cellular events responsible for the loss of cytotoxicity as shown below; they clearly differ significantly in downstream events after cytokine and receptor signaling leading to the type of cytokines secreted and the ability to modulate pro-inflammatory cytokines.

### Colocalization of cystatin F with cathepsins C and H and LysoTracker in NK92 cells

**Figure 4:**
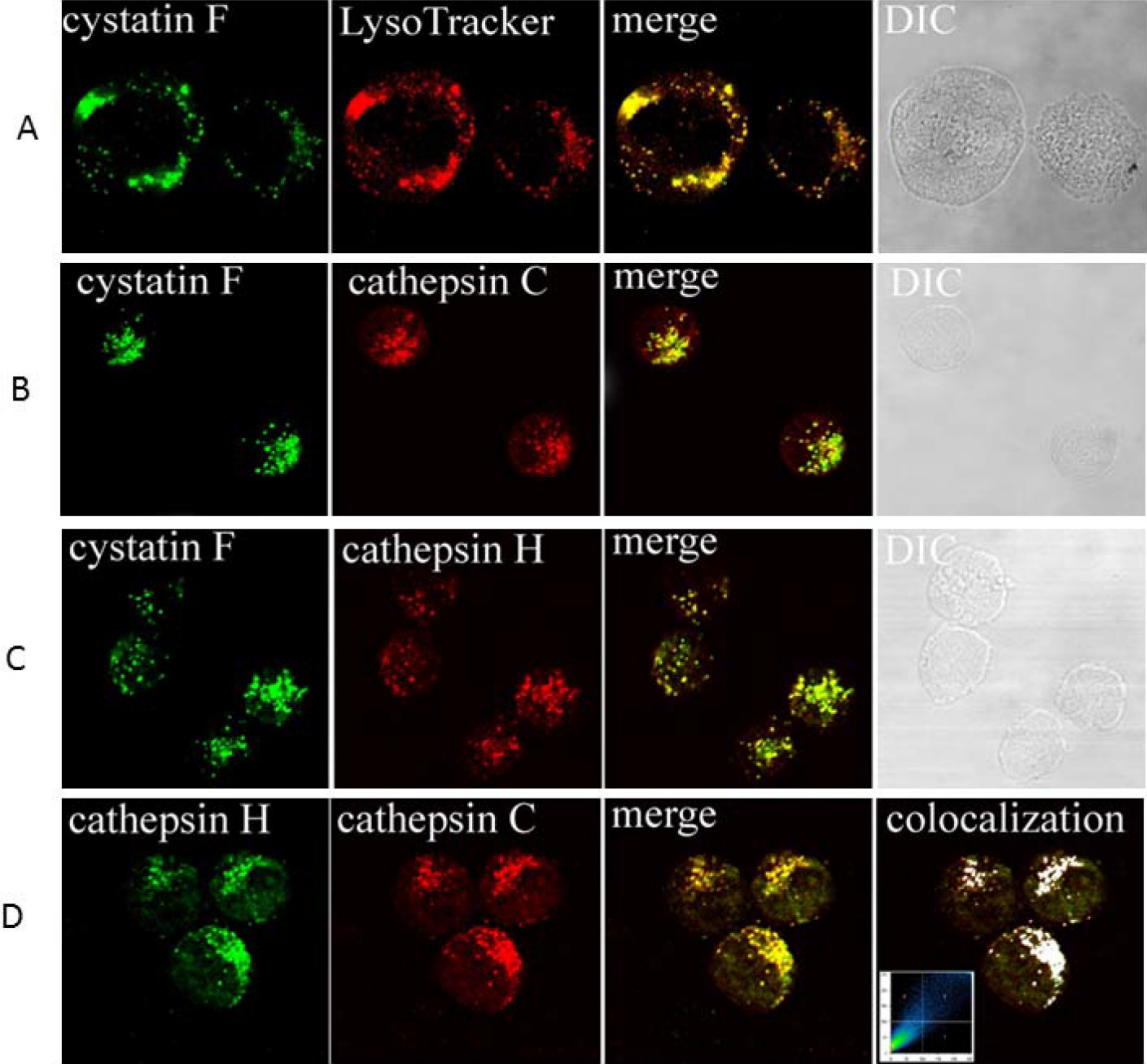
Immunofluorescence confocal microscopy: colocalization of cystatin F with LysoTracker, and cathepsin C and H in NK92 cells Colocalization of cystatin F with LysoTracker **A.** colocalization of cystatin F with cathepsin C **B.** colocalization of cystatin F with cathepsin H **C.** and colocalization of cathepsins C and H **D.** are shown. Samples were labelled with primary antibodies as indicated on micrographs: anti-cystatin F pAb **(A, B, C)**, anti-cathepsin C pAb **(B, D)**, anti-cathepsin H mAb. Red color in (A) originates from labeling with LysoTracker. Green color originates from Alexa Fluor 488-labelled secondary antibodies. Red color in **(B, C and D)** originates from Alexa Fluor 555-labelled secondary antibodies. Before merging the confocal images, signals for red and green fluorescence were adjusted to comparable levels. The yellow color indicates co-localization of two labelled antigens for cystatin F and LysoTracker **(A)**, cystatin F with cathepsin C **(B)**, and cystatin F with cathepsin H **(C)**, and cathepsins C with cathepsin H **(D)** Differential interference contrast (DIC) images are shown. In **(D)**, right image, the colocalization of both cathepsins is represented by the pixels above the threshold in both channels on the contour plot and on the merged image (white color). The relative colocalization areas, as presented by pixels in the third quadrant of the image, were higher than 40% for all co-localized pairs.

The localization of cystatin F as well as its potential targets cathepsins C and H was determined in NK92 cells. They all showed a vesicular staining, corresponding to lysosomal/endosomal vesicles. The presence of cystatin F in lysosomes was confirmed by colocalization with lysosomal marker LysoTracker (Figure 4A). Cystatin F partially colocalizes with cathepsin C (Figure 4B) and cathepsin H (Figure 4C), demonstrating both enzymes as targets for inhibitory action in NK92 cells. From Figure 4D it is evident that cathepsins C and H are colocalized in the same vesicles.

### Increased truncated monomeric cystatin F in NK92 cells

**Figure 5:**
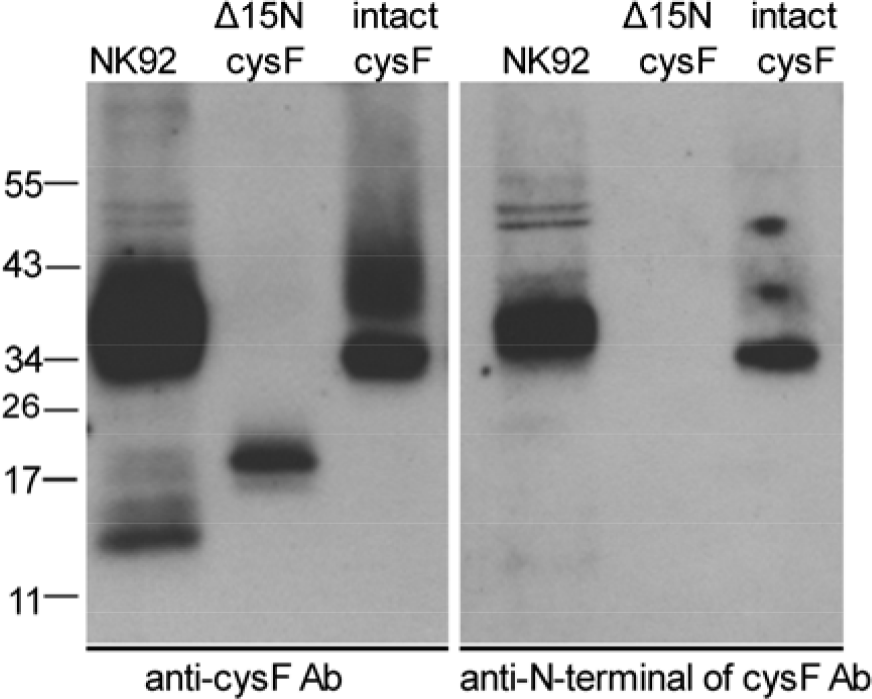
N-terminally truncated monomeric cystatin F is present in NK92 cells Lysates from NK92 cells were separated by non-reducing SDS-PAGE and blots were probed with antibodies against either whole cystatin F (anti-cys F Ab, left panel) or a 15 residue N-terminal peptide (anti-N-terminal of cysF Ab, right panel). Reactivities with recombinant intact (dimeric) cystatin F (intact cysF) and recombinant truncated (monomeric) Δ15N cystatin F (Δ15N cysF) are also shown.

As shown previously, the sequence of the cellular monomeric cystatin F isolated from U937 cells was N-terminally truncated by 15 residues to Lys35, and that the truncated but not full-length/intact monomeric cystatin F was shown to be potent inhibitor of cathepsin C [54], although the truncated form of cystatin F inhibited cathepsin H, to the similar extent as its full length/intact monomeric form. To demonstrate whether cystatin F is processed in NK92 cells, we separated the dimeric and monomeric forms of cystatin F by non-reducing SDS-PAGE and used two different antibodies for cystatin F, anti-cystatin F pAb that recognizes intact and truncated cystatin F and an antibody specific for the 15-residue N-terminal sequence of cystatin F that recognizes only intact cystatin F. We found that antibody raised against the whole cystatin F reacted with dimeric and monomeric cystatin F (Figure 5, left panel) whereas the antibody raised against N-terminal sequence of cystatin F reacted with dimeric but not with monomeric cystatin F (Figure 5, right panel). These results indicate that in NK92 cells both, monomeric and dimeric form of cystatin F is present and that the monomeric form is N-terminally truncated.

### Decreased levels of mature cathepsins C and H following addition of anti-CD16 antibody to the primary NK cells in the presence or absence of IL-2

**Figure 6:**
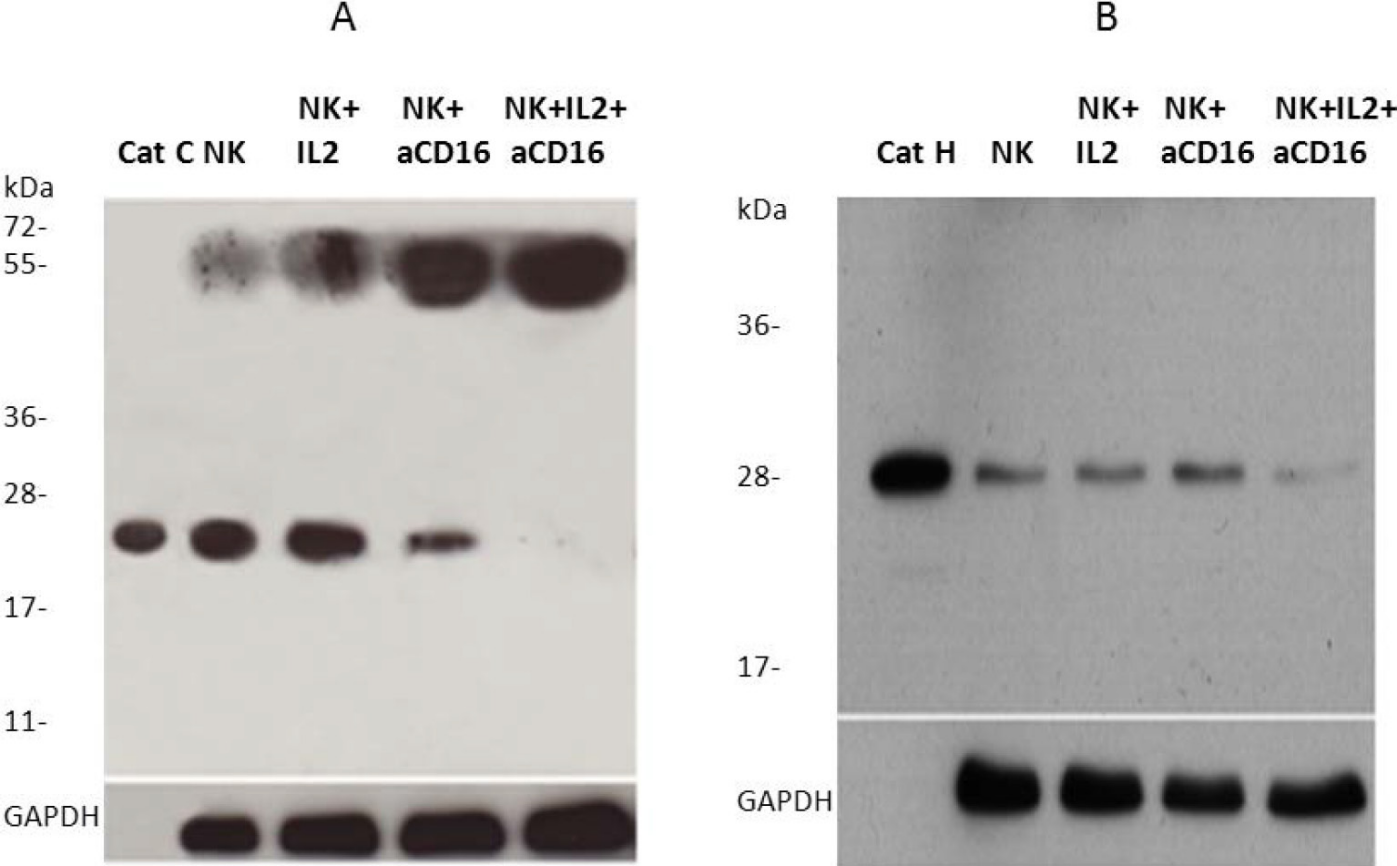
Cathepsin C and cathepsin H expression is inhibited following addition of anti-CD16 antibody to the NK cells in the presence of IL-2 1 × 10^6^ NK cells/ml were treated with IL-2 (1000 units/ml), anti-CD16 antibody (3 μg/ml), or a combination of IL-2 (1000 units/ml) and anti-CD16 antibody (3 μg/ml) for 4 hours. Lysates from NK cells were separated by reducing SDS-PAGE and blots were probed with antibodies against cathepsin C **A.** and cathepsin H **B.** GAPDH staining was used to show equivalent protein loading.

To investigate the significance and potential relationship of cathepsins C and H in the loss of cytotoxic function of primary NK cells we compared their protein levels in untreated, IL-2 treated and IL-2+anti-CD16 antibody treated NK cells (Figure 6). In the left panel (Figure 6A) the higher band (~55 kDa) corresponds to the molecular mass of pro-cathepsin C [56] and lower band (~24 kDa) to the heavy chain of mature cathepsin C [28]. Right panel (Figure 6B) represents the mature form of cathepsin H. We found that treatment of NK cells with anti-CD16 antibody with or without IL-2 significantly decreased the levels of the heavy chain of mature cathepsin C and increased the levels of pro-cathepsin C when compared to untreated and IL-2 treated NK cells, respectively (Figure 6A). For mature form of cathepsin H significant decreases were only observed when NK cells were treated with IL-2 in combination with anti-CD16mAb (Figure 6B). These results demonstrate that CD16 receptor may regulate the expression and/or processing of cathepsin C and cathepsin H. The decreased levels of both cathepsins are in line with lower cytotoxicity of NK cells, caused by anti-CD16mAb treatment.

### Increased monomeric cystatin F expression following addition of anti-CD16 antibody to the NK cells in the presence or absence of IL-2

**Figure 7:**
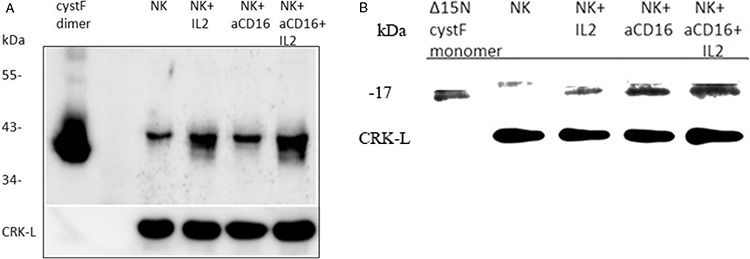
The levels of monomeric and dimeric cystatin F are increased following addition of anti-CD16mAb to the NK cells in the presence or absence of IL-2 1 × 10^6^ NK cells/ml were treated with IL-2 (1000 units/ml), anti-CD16 antibody (3 μg/ml), or a combination of IL-2 (1000 units/ml) and anti-CD16 antibody (3 μg/ml) for 4 hours. Lysates from NK cells were separated by non-reducing **A.** and reducing **B.** SDS-PAGE and blots were probed with antibodies against cystatin F. Reactivity with intact dimeric cystatin F **(A)** and truncated Δ15 monomeric cystatin F **(B)** is also shown. CRK-L staining was used to show equivalent protein loading. One of three representative experiments is shown.

To determine the expression of cystatin F in primary NK cells we compared its protein levels between untreated, IL-2 treated and IL-2+anti-CD16 antibody treated NK cells. On SDS-PAGE under non-reducing conditions we first analyzed the dimeric cystatin F in treated NK cell lysates. We found increases of dimeric cystatin F level in IL-2, anti-CD16mAb and IL-2+anti-CD16mAb treated NK cells when compared to untreated NK cells. The highest increase was seen in NK cells treated with IL-2+anti-CD16mAb (Figure 7A). On SDS-PAGE under reducing conditions, we analyzed the levels of truncated monomeric cystatin F and found significant increase of its levels in anti-CD16 treated NK cells with and without IL-2 when compared to untreated NK cells or IL-2 treated NK cells (Figure 7B). Overall, these data indicate that increased expression of monomeric cystatin F may attenuate the protease activity in anti-CD16 treated NK cells in the presence or absence of IL-2 and thus contribute to the lower cytotoxic function of primary NK cells.

### Treatment of NK cells with anti-CD16 antibody in the presence or absence of IL-2 decreased granzyme B

**Figure 8:**
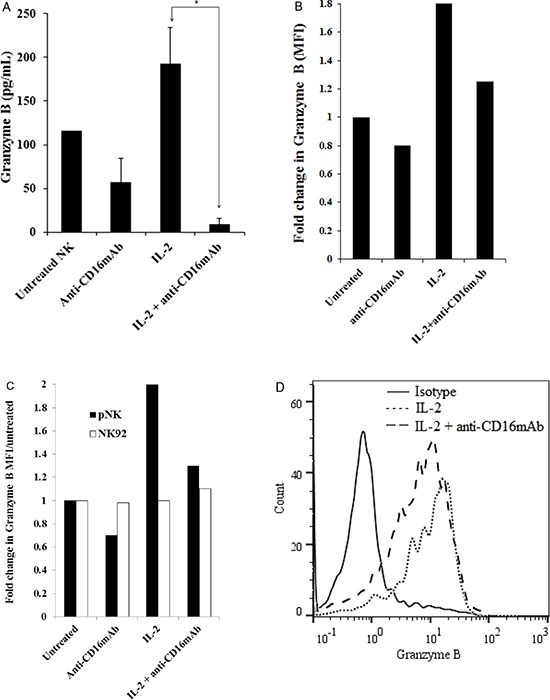
CD16 triggering in primary NK cells inhibited granzyme B Purified NK cells were treated as described in Figure 1A and after an overnight treatment, the supernatants were removed from the co-cultures and the levels of granzyme B secretions were determined using specific ELISA. *The difference between IL-2 and IL-2+anti-CD16mAb treated NK cells is significant at a *P* < 0.05 **A.** Purified NK cells were treated as described in Figure 1A and after 12–18 hours of incubation, NK cells were fixed, permeabilized and stained with PE-conjugated anti-human CD56 and FITC-conjugated anti-human granzyme B. The intracellular expression of granzyme B was assessed with flow cytometric analysis. Isotype control antibody was used as control. Mean Fluoresce Intensities (MFI) for each sample were determined and fold increase or decrease based on untreated NK cells were assessed **B.** Primary NK cells and NK92 cells (1×10^6^ cells) were left untreated or treated with anti-CD16mAb (3 μg/ml), IL-2 (1000 units/ml) or a combination of IL-2 (1000 units/ml) and anti-CD16mAb (3 μg/ml) for 24 hours before the cells were fixed, permeabilized and stained as described in the Materials and Methods section. The intracellular expression of granzyme B was analyzed by flow cytometry and Mean Fluoresce Intensities (MFI) for each sample were determined and fold increase or decrease based on untreated NK cells or untreated NK92 cells were assessed **C.** Overlay of isotype, IL-2 treated NK cells and IL-2+anti-CD16mAb treated NK cell histograms were analyzed by FlowJo software **D.**

To determine the relationship between cytotoxicity, cathepsin C and H expression and cystatin F levels in NK cells, we determined the granzyme B, a final executive molecule in NK cell cytotoxicity in untreated, IL-2 treated and IL-2+anti-CD16 antibody treated NK cells. Our results show that granzyme B protein level is increased in IL-2 treated NK cells whereas it is decreased in NK cells treated with anti-CD16mAb with and without IL-2 correlating with decreased cytotoxicity of NK cells against target cells as assessed by ELISA (Figure 8A). Similarly, a significant decrease in granzyme B expression is seen when untreated, anti-CD16mAb treated and/or IL-2 treated NK cells are cultured with OSCSCs as compared to those in the absence of OSCSCs (data not shown). Similar results to those seen with ELISA were also observed when granzyme B levels were determined by flow cytometric analysis in NK cells (Figures 8B and 8D). There was approximately 2 fold increase in mean fluorescence intensity of granzyme B in IL-2 treated primary NK cells when compared to untreated NK cells whereas there was a decrease in anti-CD16mAb and IL-2+anti-CD16mAb treated NK cells (Figure 8B). The levels of granzyme B remained very low in NK92 cells and no differences can be seen in any of treated NK92 cells when compared to untreated cells (Figure 8C).

## DISCUSSION

The interaction of NK cells with primary oral tumor cells was studied after treatment with IL-2 and/or anti CD16 antibody with and without monocytes. IL-2 treated primary NK cells were cytotoxic to oral tumor stem cells whereas anti-CD16 antibody treatment decreased untreated or IL-2 treated NK cell cytotoxicity significantly due to the induction of split anergy in NK cells. In contrast to primary NK cells, IL-2 treated NK92 cells mediated no/low cytotoxicity against OSCSCs and induced no/very low secretion of IFN-γ and TNF-α and high levels of IL-6 and IL-10. NK92 cells expressed much lower levels of CD16 and NKp46 but higher levels of NKp44 when compared to primary NK cells. NK92 cells remained unresponsive to signals from anti-CD16mAb and bacteria and did not increase cytotoxicity or IFN-γ secretion even in the presence of CD16 receptor transfection and surface expression, indicating the potent and perhaps irreversible anergic nature of these cells. Antibody to IL-10 did not change the levels of IFN-γ secretion in NK92 cells whereas it significantly increased IFN-γ secretion in primary NK cells. Split anergized primary NK cells significantly upregulated IFN-γ secretion in the absence of cytotoxicity when treated with IL-2+anti-CD16mAb cultured with and without monocytes. Because of the lack of cytotoxicity in NK92 cells and split anergized primary NK cells we determined the levels and expression of upstream regulators of granzyme B, a known executioner of cytotoxicity in NK cells. We demonstrate in this paper that active form of aminopeptidases cathepsins C and H are significantly down-modulated by CD16 receptor signaling due to the lower processing of their pro-peptides into active enzymes, which is likely due to the increased expression and function of their endogenous inhibitor, cystatin F.

Target-induced inactivation of NK cytotoxicity and induction of split anergy in NK cells due to CD16 receptor triggering has been shown previously [4, 5]. Loss of CD16 receptor expression and NK cell cytotoxic function were also seen in many cancer patients indicating the physiological relevance of our observation in cancer patients [10, 11]. The precise mechanism of split anergy in NK cells is not known completely and is the subject of this paper, however it includes apoptosis in a small subset of NK cells, indicating the action of caspases contributing in part to this process [4, 5, 7, 57].

To further delineate and identify upstream regulators of split anergy in NK cells, we determined the levels of active cathepsin C and H and their inhibitor cystatin F in split anergized primary NK cells and in NK92 cells. When activated with IL-2, NK92 cells were found to have no/low cytotoxicity against NK sensitive cancer stem cells; however, they retained the ability to secrete high levels of regulatory cytokines IL-6 and IL-10 with no or slight secretion of IFN-γ and TNF-α. Therefore, although there are significant differences between split anergized primary NK cells and NK92 cells in regards to the type of cytokines they secrete, they share upstream signaling mechanisms for the loss of cytotoxicity. NK92 cells do not express CD16 receptors and are unable to increase the expression of CD16 receptors when treated with IL-2 (data not shown). Similarly, tumor and anti-CD16mAb mediated split anergized NK cells lose CD16 receptors and addition of IL-2 does not fully reverse down-modulation of CD16 receptor (data not shown). In addition, similar to split anergized NK cells, IL-2 treatment does not increase granzyme B expression on NK92 cells, whereas they increase secretion of both IL-10 and IL-6. OSCSCs induced decrease in granzyme B expression and secretion similar to anti-CD16mAb mediated decrease is not reversed when treated with IL-2. Therefore, there is a dissociation between cytotoxicity and cytokine secretion within split anergized NK cells and NK92 cells, and NK92 cells can be used to study the upstream regulators of cytotoxicity even though the profiles of cytokine secretion and their regulations may differ between the two model systems. Indeed, NK92 cells and split anergized primary NK cells share similarities in the function of several upstream regulators of cytotoxicity as described below. Differences in the regulation of cytokines can also be seen between split anergized primary NK cells and NK92 cells. NK92 cells have lost the ability to respond to Toll Like Receptors TLRs since they do not respond to bacterial triggering, whereas split anergized NK cells respond greatly to bacteria by upregulating both pro-inflammatory and anti-inflammatory cytokines. This could be due to the loss of TLRs on NK92 cells. NK92 cells do not increase IFN-γ secretion when treated with anti-IL10 antibodies or bacteria, whereas split anergized NK cells substantially increase IFN-γ when treated with anti-IL10 antibodies or treated with bacteria while exhibiting no or decreased cytotoxicity. Dissociation between NK cytotoxicity and cytokine secretion has also been observed by other investigators [58, 59].

We demonstrate much lower expression of heavy chain of mature cathepsin C (with molecular weight of 24 kDa) in split anergized NK cells with low cytotoxicity when compared to non-treated or IL-2 treated NK cells. The results also indicate that triggering of CD16 receptor on NK cells decreases the processing of pro-cathepsin C, to mature cathepsin C and thus induces the accumulation of pro-cathepsin C. Decrease in active cathepsin C should result in decreased processing and lower activation of granzyme B [29, 34]. Cathepsin H is an additional convertase of pro-granzyme B which is inhibited in split anergized primary NK cells. By confocal microscopy we identified cathepsins C and H in the same endosomal/lysosomal vesicles in NK92 cells confirming previously suggested functional redundancy of these two enzymes in granzyme B activation.

Cystatin F expression is limited to immune cells such as monocytes, T cells, NK cells and dendritic cells (DCs) [48, 50, 60, 61]. The 29-fold increase in gene expression for cystatin F was shown in human NK cells compared to CD8^+^ T lymphocytes [61]. Cystatin F is present intracellularly in active monomeric and inactive dimeric forms, whereas secreted cystatin F is predominantly in the form of inactive dimer [45, 52, 62]. It was shown that in promonocyte U937 cells, a large proportion of the cystatin F resides in the endosome/lysosome-like vesicles [44, 45] as a truncated monomeric form, whereas in ER and Golgi apparatus inactive dimeric form predominates [54]. Similarly, our results demonstrate lysosomal localization of cystatin F in NK92 cells. Moreover, the monomeric fraction of cystatin F in NK92 cells is in a truncated form that is able to inhibit cathepsins C and H. Similarly, the primary NK cells also possess truncated monomeric cystatin F along with inactive dimer. Cathepsin C has been suggested as the main target of cystatin F in cytotoxic cells such as T cells and NK cells [29, 54, 63]. Partial co-localization of cystatin F and cathepsin C in NK92 cells supports this hypothesis. Additionally, increased expression of truncated monomeric cystatin F in primary NK cells with reduced cytotoxicity favors its potential in regulation of cathepsin C activity and the cytotoxic function. Cathepsin H represents another target for inhibition by cystatin F. Truncated monomeric cystatin F inhibits cathepsin H in a nanomolar range (Ki 8.3 ± 4.7 nM, [46]) and may prevent the generation of active granzyme in vesicles or cells lacking the activity of cathepsin C. Cystatin F is able to inhibit cathepsin H also as an intact monomer, however, in this study we did not determine intact monomeric form of cystatin F in cell lysates of NK cells. Increased levels of cystatin F in NK cells could be as a result of accelerated synthesis and/or internalization of secreted cystatin F. For the latter, it has been shown that extracellular dimeric cystatin F could be internalized and then activated in endosomal/lysosomal vesicles within the cells [64]. One may speculate that target cells may secrete inactive cystatin F which after internalization and activation may inhibit cathepsins C and H and down-regulate NK cell cytotoxicity. Indeed, significant inhibition of granzyme B expression was evident in NK cells after their culture with the OSCSCs, and no increase can be seen with any of the NK cell treatment.

In parallel with the lower levels of cathepsin C and H and higher levels of truncated monomeric cystatin F in NK92 cells and split anergized primary NK cells, we observed significant decreases in granzyme B expression correlating with loss of cytotoxicity in NK cells. As shown in our previous study, split anergized NK cells also demonstrated decreased levels of mRNA for perforin, granzyme A and granzyme B [8]. Interestingly, we also observed significant decreases for granulysin in split anergized NK cells (data not shown). In agreement with our findings on anti-CD16 mAb split anergized NK cells, Hanna et al demonstrated 2 fold decrease in granzyme B gene expression in CD16-CD56+ NK cell subset when compared to CD16^+^CD56 ^dim^ subset [65].

Overall, our results demonstrate that the induction of functional split anergy in NK cells by anti-CD16 antibody decreases the levels of mature effector cathepsins C and H, the key molecules involved in granzyme B activation responsible for granule-mediated target cell lysis. The activities of cathepsins C and H are likely inhibited by the higher levels of monomeric cystatin F, a cysteine protease inhibitor, capable of acting in the endosomal/lysosomal vesicles in cytotoxic cells. Therefore, our studies identify and characterize several upstream targets/regulators of split anergized NK cells, paving the road for the future mechanistic studies to restore the cytotoxic function of anergized NK cells. In addition, contribution of other related proteases in the induction of split anergy should also await future studies.

## MATERIALS AND METHODS

### Cell lines and reagents

RPMI 1640 supplemented with 10% FBS was used to culture human NK cells and human PBMCs. NK92 cells were obtained from ATCC and were maintained in RPMI 1640 supplemented with 12.5% Fetal Bovine Serum (FBS), 12.5% horse serum and 100 units/mL IL-2. NK92 and its CD16 transfectants were generous gift from Dr. Kerry Campbell. Oral Squamous Carcinoma Stem Cells (OSCSCs) and differentiated Oral Squamous Carcinoma Cells (OSCCs) were isolated from resected tongue tumors at UCLA, and were cultured in RPMI 1640 supplemented with 10% FBS and maintained in the laboratory for long term use. Recombinant IL-2 was obtained from NIH-BRB. Single ELISA kits were purchased from Biolegend (San Diego, CA). Fluorokine MAP cytokine multiplex kits were purchased from R&D Systems (Minneapolis, MN). sAJ2 is a combination of eight sonicated gram positive probiotic bacteria selected for their superior ability to induce optimal secretion of both pro-inflammatory and anti-inflammatory cytokines in NK cells (manuscript in prep).

### Antibodies and proteins

Rabbit anti-cystatin F polyclonal antibody, recombinant cistatin F and mouse 1D10 anti-cathepsin H Mab was prepared by our group in Ljubljana as reported [45, 50]. Rabbit N1 antibody against N-terminal of cystatin and Δ15N cystatin F were prepared at Dundee University, Dundee, UK, [54]. Goat anti-cathepsin C pAb was from R&D Systems (Minneapolis, MN), mouse anti-GAPDH mAb was from Invitrogen and rabbit anti-CRK-L pAb was from Sigma. The dye identifying the lysosomes, LysoTracker, was from Molecular Probes (Eugene, OR). Secondary labelled antibodies goat anti-rabbit Alexa Fluor 488 and donkey anti-goat Alexa Fluor 555 were from Molecular Probes (Eugene, OR). Antibodies to CD16 were purchased from Biolegend (San Diego, CA). FITC anti-human/mouse granzyme B antibody was purchased from Biolegend (San Diego, CA).

### Purification of NK cells and monocytes

Written informed consents approved by UCLA Institutional Review Board (IRB) were obtained from the blood donors and all the procedures were approved by the UCLA-IRB. PBMCs and NK cells from healthy donors were isolated as described [4]. Briefly, peripheral blood lymphocytes were obtained after Ficoll-hypaque centrifugation and purified NK cells were negatively selected by using NK cell isolation kit (Stem Cell Technologies, Vancouver, Canada). The purity of NK cell population was found to be greater than 90% based on flow cytometric analysis of anti-CD16 antibody labelled cells. The levels of contaminating CD3^+^ T cells remained low, at 2.4% ± 1%, similar to that obtained by the non-specific staining using isotype control antibody throughout the experimental procedures. The adherent subpopulation of PBMCs was detached from the tissue culture plates and monocytes were purified using isolation kit obtained from Stem Cell Technologies (Vancouver, Canada). Greater than 95% purity was achieved based on flow cytometric analysis of CD14 antibody labeled monocytes.

### ^51^Cr release cytotoxicity assay

The ^51^Cr release assay was performed as described [15]. Briefly, target cells were either co-cultured or not with irradiated (10 Gy) monocytes for 24–48 hours before they were labeled with ^51^Cr for 1 hour, when they were washed and added at different ratios of NK cells. After 4 hour incubation, the supernatants were harvested from each sample and counted for released radioactivity using the γ counter. The purified NK cells were either left untreated or treated with anti-CD16mAb (3 μg/ml), IL-2 (1000 units/ml), or a combination of IL-2 (1000 units/ml) and anti-CD16mAb (3 μg/ml) for 24–48 hours before they were added to ^51^Cr labeled target cells. The percentage of cytotoxicity was calculated as follows:

% Cytotoxicity = (experimental cpm − spontaneous cpm)/(total cpm − spontaneous cpm)

LU 30/10^6^cells were calculated using the inverse of the number of effector cells needed to lyse 30% of target cells × 100.

### ELISA and multiplex assays

Single ELISAs were performed as described previously [66]. Fluorokine MAP cytokine multiplex kits were conducted as suggested by the manufacturer. To analyze and obtain the cytokine and chemokine concentration, a standard curve was generated by either two or three fold dilution of recombinant cytokines provided by the manufacturer. Analysis was performed using the Star Station software. Samples were analyzed using Beckman Coulter EPICS XL cytometer and subsequently analyzed in FlowJo software (Tree Star).

### Intracellular stain and flow cytometry

NK cells were fixed and permeabilized with Fixation Buffer and Permeabilization Buffer purchased from Biolegend (San Diego, CA) and the procedures were conducted as instructed by the manufacturer.

### Western blot

1 × 10^6^ NK cells/ml were treated with IL-2 (1000 units/ml), anti-CD16 antibody (3 μg/ml), or a combination of IL-2 (1000 units/ml) and anti-CD16 antibody (3 μg/ml) for 4 h. NK92 cells were cultured in complete RPMI 1640 medium. Cells were washed twice and lysed by the lysis buffer (150 mM NaCl, 1% (v/v) Triton X-100, 2 mM EDTA, 100 mM citrate buffer pH 5.5) with the addition of 2 mM phenylmethylsulfonyl fluoride (Sigma), 10 μg/ml leupeptin (ICN, Aurora, OH), 2 units/ml aprotinin (ICN, Aurora, OH) for 20 min on ice. The samples were then centrifuged at 10,000 rpm for 10 min at 4°C to remove the nuclei, the supernatants were removed and the levels of proteins quantified by the Bradford method. The lysates were boiled in 4 × SDS sample buffer and reduced with the addition of 200 mM DTT. Samples were loaded onto 12% SDS-PAGE and gels immunoblotted to a Immobilon-P membranes (Millipore, Billerica, MA). The membranes were blocked with 5% non-fat milk in PBS plus 0.1% Tween-20 for 1 hour and incubated in predetermined dilutions of primary antibodies overnight at 4°C. Membranes were then incubated corresponding horseradish peroxidase-conjugated secondary antibody. Blots were developed by enhanced chemiluminiscence (ECL-purchased from Pierce Biotechnology, Rockford, IL). Density of protein bands was analysed by Image J. Relative density values were calculated by dividing the percent values of each sample by the percent value of the contol (untreated NK). Further, adjusted denstity values were obtained by scaling the relative density values for the samples by the relative density values of the corresponding loading control bands for each lane.

### Confocal immunofluorescence microscopy

NK92 cells (1 × 10^5^) were centrifuged with cytospin (Cytofuge) for 6 min at 1000 rpm onto glass cover slides. Before labelling, cells were allowed to recover for 15 min and then fixed with 4% paraformaldehyde for 45 min and permeabilized by 0.1% Triton X-100 in PBS, pH 7.4, for 10 min. Nonspecific staining was blocked with 3% BSA in PBS, pH 7.4, for 1 hour. Lysosomes were labelled by adding LysoTracker to the cells for 2 min before fixation with 4% paraformaldehyde, as recommended by Molecular Probes. All primary antibodies were added after nonspecific blocking. After 2 hours incubation with primary antibody, cells were washed with PBS and treated with fluorophore-labelled secondary antibody for 1 hour. After a final wash with PBS, the ProLong antifade kit (Molecular Probes) was used to mount coverslips on glass slides. Control samples were run in the absence of primary antibodies. Fluorescence microscopy was performed using a Carl Zeiss LSM 510 confocal microscope with 63x Plan-Apochromat Oil DIC objective, N.A. 1.4. Alexa Fluor 488, LysoTracker, or Alexa Fluor 555 was excited with an argon (488 nm) or He/Ne (543 nm) laser, and emission was filtered using narrow-band LP 505–530 nm (green fluorescence) and LP 560 nm (red fluorescence) filters, respectively. Images were analyzed using Carl Zeiss LSM image software 3.0.

### Statistical analysis

An unpaired, two-tailed Student *t*-test was used to analyze the differences between the samples. A *p*-value of < 0.05 was considered statistically significant.
